# Factores socioeconómicos y zona de residencia como estratificadores de desigualdades en salud en Bolivia

**DOI:** 10.26633/RPSP.2017.155

**Published:** 2017-11-11

**Authors:** Wilson René Alarcon

**Affiliations:** 1 Organización Panamericana de la Salud Bolivia La Paz Bolivia Organización Panamericana de la Salud, La Paz, Bolivia.

**Keywords:** Healthcare disparities, health inequalities, socioeconomic factors, Disparidades en atención de salud, desigualdades en la salud, factores socioeconómicos, Disparidades em assistência à saúde, desigualdades em saúde, fatores socioeconómicos

## Abstract

**Objetivo.:**

*Describir las desigualdades en salud respecto a indicadores de cobertura estratificado por factores socioeconómicos y lugar de residencia*.

**Métodos.:**

*Se realizó un estudio ecológico con datos de las Encuestas de Demografía y Salud 2003, 2008 y la Encuesta Nacional de Salud y Nutrición 2012. Con un análisis de 15 variables respecto a estratificadores socioeconómicos y de lugar de residencia*.

**Resultados.:**

*El Índice Compuesto de Cobertura (ICC) calculado mostró que los grupos más pobres se han acercado a los grupos más ricos reduciendo la brecha de desigualdad, el cambio en los quintiles más ricos no ha sido tan acelerado como en los quintiles más pobres. Varios de los indicadores han tenido avances más acelerados para el grupo más pobre como el de partos atendidos por personal de salud, otros indicadores han presentado condiciones de crecimiento reducido en la disminución de la desigualdad. La desnutrición crónica en niños menores de 5 años ha disminuido, sin embargo, la anemia en el mismo grupo de edad se ha incrementado*.

**Conclusiones.:**

*Los indicadores mostraron avances en la reducción de la desigualdad y disminución en la brecha entre los grupos más pobres y los más ricos. El uso de métodos de planificación familiar, por la presencia de bonos de transferencia condicionada podría no haber logrado el crecimiento esperado. Se deben evaluar estrategias como la suplementación con hierro a niños menores de 5 años, que ha crecido en la provisión del suplemento, pero no ha tenido impacto en la reducción de la anemia*.

## Abstract (en)

**Objective.:**

Describe health inequalities with regard to coverage indicators stratified by socioeconomic factors and place of residence.

**Methods.:**

An ecological study was conducted with data from the 2003 and 2008 Demographic and Health Surveys and the 2012 National Health and Nutrition Survey, with analysis of 15 variables for socioeconomic and place-of-residence stratifiers.

**Results.:**

Calculation of the composite coverage index showed that the poorest groups have moved closer to the richest groups, bridging the inequality gap; change in the richest quintiles has not been as rapid as in the poorest quintiles. Several of the indicators have seen more rapid progress in the poorest group, such as that for deliveries attended by health personnel; other indicators showed reduced growth in reduction of inequality. Chronic malnutrition in children under 5 has decreased; however, anemia in this age group has increased.

**Conclusions.:**

The indicators showed advances in reduction of inequality and reduction in the gap between the poorest and richest groups. Expected growth in the use of family planning methods may not have been reached because of the existence of conditional cash transfers. Strategies should be evaluated, such as iron supplementation in children under 5, where provision of the supplement has increased, but it has not had an impact on anemia reduction.

## RESUMO

**Objetivo.:**

Descrever as desigualdades em saúde segundo indicadores de cobertura estratificados por fatores socioeconômicos e área de residência.

**Métodos.:**

Foi realizado um estudo ecológico com dados obtidos das Pesquisas de Demografia e Saúde de 2003 e 2008 e da Pesquisa Nacional de Saúde e Nutrição de 2012 baseado na análise de 15 variáveis relacionadas a variáveis de estratificação por fatores socioeconômicos e área de residência.

**Resultados.:**

O índice de cobertura composto (ICC) calculado demonstrou que os estratos de menor renda estão mais próximos aos estratos de maior renda, diminuindo o hiato de desigualdade, mas a mudança nos quintis de maior renda não foi tão acelerada como nos quintis de menor renda. Observou-se progresso mais acentuado em vários indicadores no estrato de menor renda como o número de partos assistidos por profissionais da saúde, mas pouco avanço em outros indicadores para reduzir as desigualdades. Em crianças menores de cinco anos, o número de casos de desnutrição crônica diminuiu, mas de anemia aumentou.

**Conclusões.:**

Os indicadores apontam avanços na redução da desigualdade em saúde, com menor hiato entre os estratos de menor e maior renda. O uso de métodos de planejamento familiar, como parte de programa de transferência de renda condicionada, possivelmente não teve o crescimento esperado. Algumas estratégias precisam ser reavaliadas, por exemplo a suplementação de ferro em crianças menores de cinco anos: houve um aumento na provisão de ferro, porém ele não surtiu efeito na redução da anemia.

En el año 2013, la Organización Mundial de la Salud (OMS), dejó claro que las desigualdades en salud “*esas diferencias en salud observables e injustas entre personas de grupos sociales diferentes, y que son resultado de formas de desventajas como la pobreza, la discriminación y la falta de acceso a servicios y de la distribución de los recursos y bienes*” si pueden ser medidas y monitoreadas y sirven como un medio indirecto de evaluar la inequidad en salud, que se trata de un concepto más normativo y que depende del contexto político y el concepto de justicia social de cada país (1).

Bolivia, debe iniciar la medición de los Objetivos de Desarrollo Sostenible *(ODS)*, en este contexto, el monitoreo de las desigualdades en salud cobra relevancia para describir las diferencias y cambios entre grupos, que aporte a la toma de decisiones en políticas y programas.

El informe *Countdown to 2015* (2) hace mención a los avances logrados en la disminución de las desigualdades en salud en Bolivia. Los datos utilizados para elaborar ese informe están basados en la Encuesta Nacional de Salud *(ENDSA)* 2008 (3, 4), que es hasta el momento, la última publicación disponible en el país.

Con el propósito de aportar mayor evidencia de los posibles cambios sucedidos en los periodos más recientes, el presente manuscrito, incorporó información publicada de la Encuesta Nacional de Salud y Nutrición *(ESNUT)* llevada a cabo en el año 2012 (5), esta encuesta posee representatividad nacional, con variables estándar y un nivel de desagregación suficiente que permite la comparación con las ENDSA 2003 (6) y 2008.El objetivo del presente documento es describir las desigualdades en salud respecto a indicadores de cobertura, estratificado por factores socioeconómicos y lugar de residencia; relacionado a indicadores de cobertura, desnutrición crónica y presencia de anemia en niños menores de 5 años, lo que permitirá mostrar si hubo cambios entre los periodos analizados.

## MATERIALES Y MÉTODOS

### Diseño del estudio y fuentes de datos

Se realizó una comparación de tres encuestas en salud desarrolladas en Bolivia: las Encuestas de Demografía y Salud 2003, 2008, y la Encuesta de Salud y Nutrición 2012; las mismas utilizaron metodologías estándar de medición, lo que permitió el cálculo de indicadores en salud similares a los monitoreados y publicados por *Countdown to 2015*.

El tipo de estudio desarrollado fue ecológico (7), con fuente de datos secundarios que miden indicadores en salud en los periodos 2003, 2008, y 2012, este tipo de estudio ha facilitado la comparación entre las tres encuestas.

### Variables dependientes

Se realizó la comparación de la cobertura de intervenciones en salud reproductiva, materna, neonatal e infantil, que representa la provisión de servicios y el uso de servicios de salud para una población determinada (Bolivia), respecto a clasificadores de pobreza y zona de residencia.

Las Encuestas Nacionales de Demografía y Salud 2003 y 2008 elaboradas con base a muestras probabilísticas con representatividad nacional, estratificadas por conglomerados bietapicos, con probabilidades proporcionales a su tamaño (autoponderadas).

La Encuesta de Salud y Nutrición 2012, con un diseño similar a las ENDSA, estratificada con probabilidades de selección proporcionales a su tamaño por conglomerados bietapicos.

Las unidades de análisis fueron las mujeres en edad fértil y niños menores a cinco años en los hogares seleccionados. La cobertura en salud se midió a través de un índice que resumió las diferentes intervenciones en el sistema de salud (8), este incluye ocho indicadores del continuo de atención, que es la atención sistematizada a las personas en el curso de vida en el contexto de un sistema de salud estructurado.

Se planteó el Índice Compuesto de Cobertura *(ICC)* como variable dependiente, utilizado en comparaciones entre países y análisis de equidad (9,10) y da igual peso a cuatro etapas del continuo de atención: a) planificación familiar; b) atención materna y neonatal; c) inmunización; y d) manejo de casos de niños enfermos (8,11). El cálculo del índice compuesto de cobertura (ICC) ha sido definido a partir de la siguiente fórmula:

$$ICC=\frac{(NPFS)+\left(\frac{APN+PPS}{2}\right)+\left(\frac{2*PENTA3+BCG+SRP}{4}\right)+\left(\frac{TRO+FE}{2}\right)}{4}$$

Donde^2^:

NPFS: necesidad de planificación familiar satisfecha

PENTA3: pentavalente en su tercera dosis

BCG: vacuna contra la tuberculosis SRP: vacuna contra el sarampión – rubeola – paperas

APN: atención prenatal por personal de salud

PPS: atención de parto por personal de salud

TRO: tratamiento de rehidratación oral y continuación de alimentación

FE: suplemento de hierro

Se realizaron cambios al ICC con respecto al estudio *Countdown* 2015: se reemplazó el indicador de búsqueda de atención por neumonía (no incluido el 2012) incorporando el tratamiento otorgado para sospecha clínica de anemia, es decir la provisión de suplementos de hierro a niños de 6 a 59 meses. La elección de esta variable se hizo sustentado en las políticas más recientes del país, que durante los últimos años ha hecho hincapié en esta estrategia, para la descripción detallada de indicadores y datos ver anexos.

El indicador de necesidades satisfechas de planificación familiar para la ESNUT 2012 se sustituyó por el valor de la ENDSA 2008, cambio que se sustentó en procedimientos metodológicos ya realizados en otros estudios (12).

Se utilizaron los resultados para la vacuna pentavalente en su tercera dosis y no DPT3.

Con estos ajustes se diseñó y aplicó el Índice Compuesto de Cobertura *(ICC) (13)*.

En este estudio también se analizaron dos indicadores de impacto: prevalencia de retraso en el crecimiento en niños menores de 5 años, y prevalencia de anemia.

### Variables independientes

Como variables independientes se utilizaron una socioeconómica y otra de zona de residencia; la primera se analizó a través de la desagregación por quintiles de riqueza de los hogares (datos disponibles en las tres encuestas) que se basa en la construcción de índices de activos del hogar, características de la vivienda e infraestructura disponible (14).

Los quintiles de riqueza son grupos poblacionales que contienen al 20% de la población encuestada en cada grupo, siendo el quintil 1 el que representa al grupo con mayor pobreza, y el quintil 5 el que representa a los más ricos, de acuerdo al índice de riqueza; no se incluye variables de ingresos o egresos económicos para la construcción de este índice (15).

La zona de residencia se clasificó como residencia urbana y residencia rural, de acuerdo a caracterización establecida por el Instituto Nacional de Estadística.

### Análisis de datos

La inclusión de la ESNUT como fuente de datos, mejoró la capacidad de análisis y monitoreo de varios indicadores, ya que en el país existe muy poca información con características de estudios a nivel nacional que realice una clasificación en quintiles de riqueza a partir de la medición de indicadores socioeconómicos, tener esta información en los tres estudios permitió realizar la comparación.

Se analizaron 15 indicadores considerados de relevancia; 13 de ellos muestran coberturas en procesos de atención o prevención en salud y dos muestran impactos en niños menores de 5 años.

### Medición de las desigualdades

Se realizó el cálculo de dos índices: el índice de desigualdad de la pendiente (IDP) y el índice de concentración (IC) que permiten explicar el comportamiento de los indicadores en el tiempo.

Los cálculos para ambos índices se realizaron a partir de datos agrupados, en el software Epidat 4.2.

El índice de concentración (IC), expresa la desigualdad relativa, muestra cuán lejos de la igualdad se encuentra determinada intervención o servicio en una escala de (-100 a 100), con cero representando la equidad total, medido sobre el índice de riqueza (16). Los valores positivos se encuentran generalmente en las variables de cobertura de salud, mostrando que los más ricos tienen mejores accesos a los servicios de salud, mientras que las distribuciones negativas se presentan en las variables de impacto, como anemia, retraso en crecimiento o mortalidad.

El índice de desigualdad de la pendiente (IDP), expresa la desigualdad absoluta, que es básicamente la pendiente de la recta de regresión (*b*) que toma en consideración la situación socioeconómica de los grupos como el tamaño de la población, es decir *y=a+bx*, donde la variable *y* es la variable de salud y *x* es el valor *ridit* del grupo ordenado por la función de la variable socioeconómica (17). Se estima por el método de los mínimos cuadrados ponderados, donde el factor de ponderación es el tamaño poblacional total del país en cada uno de los quintiles de riqueza y representa la diferencia entre los puntos extremos de la escala con respecto a la variable de salud, varía entre 0 y 100%. Si es negativa, las dos variables varían en direcciones opuestas (18).

### Ética

Todos los análisis están basados en datos disponibles de encuestas nacionales publicadas, las autorizaciones éticas fueron responsabilidad de las instituciones que administraron las encuestas. El presente estudio utiliza datos agrupados y no individualiza participantes.

## RESULTADOS

El análisis comparó un total de 13 indicadores de cobertura en salud y dos indicadores de impacto de desnutrición crónica y anemia en niños menores de 5 años (anexo 2).

**FIGURA 1.**
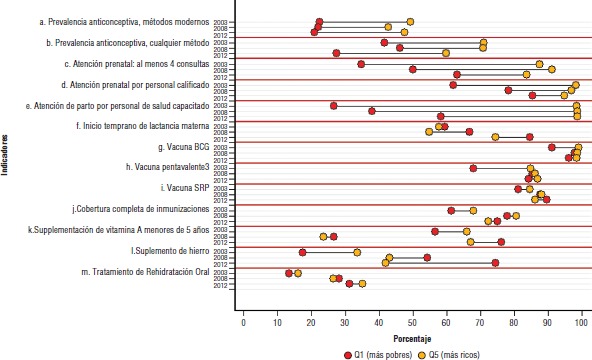
Indicadores de cobertura estratificados según niveles de riqueza por año. Bolivia

Una visión general de las desigualdades se grafican en la figura 1, mostrando todas las variables según los quintiles de riqueza extremos, donde están representados los más pobres (Q1 rojo), y los más ricos (Q5 naranja). Las distancias más amplias entre Q1 y Q5 determinan una desigualdad mayor sea pro-rico en el caso de estar el naranja a la derecha (ej. atención prenatal 4 controles) o pro-pobre cuando esté el rojo más a la derecha del gráfico (ej. suplemento de hierro 2008 y 2012).

***Fuente:*** elaboración propia, datos ENDSA 2003, 2008 y ESNUT 2012.

**Nota:** las líneas horizontales conectan los quintiles más pobres (círculos rojos) y los más ricos (círculos dorados); Cuanto más larga sea la línea entre los dos grupos, mayor será la desigualdad absoluta; los puntos representan las coberturas promedio en cada quintil de riqueza.

### Desigualdades en cobertura respecto a quintiles de riqueza

Los datos de la figura 1 muestran en el eje de las Y los indicadores de salud y en el eje de las X el porcentaje para cada indicador, estos sugieren que persisten o se han incrementado las desigualdades en cuanto a la planificación familiar, donde la prevalencia de anticoncepción con métodos modernos no ha sufrido modificaciones significativas, con variaciones inferiores al 5% entre los años 2003 y 2012 y la anticoncepción con el uso de cualquier método ha disminuido, siendo en el 2008 15,5% más alto el promedio nacional que en el 2012, este descenso se presentó en todos los quintiles de riqueza y en área urbana y rural.

La cobertura en la atención prenatal por personal de salud se ha incrementado para Q1 en 23,4% en el periodo 2003 – 2012 (figura 1). La desigualdad absoluta, ha disminuido en 31,7% (42,^2^-^12^,^5^) puntos porcentuales y las desigualdades relativas mantienen una distribución pro-rica, pero con niveles más cercanos a la paridad.

Los datos insinúan un crecimiento en el promedio nacional de cobertura del parto atendido por personal de salud en 24,3% entre el periodo 2003 – 2012, el mayor incremento se registra en Q1 con 31,7% de crecimiento respecto a 0,2% en Q5. La desigualdad absoluta ha disminuido de 89,8 a 57,9 (31,9 puntos) entre el 2003 y el 2012 y el índice de concentración que aún sigue siendo positivo disminuyó de 23,5 a 11,5.

Las coberturas reproductivas y maternas han tenido menos logros en cuanto a la disminución de las desigualdades que las administradas a los niños menores de 5 años.

El inicio temprano de la lactancia materna, con mejores niveles para los grupos más pobres, presenta un incremento en el promedio nacional para el 2012. Este indicador es peculiar, ya que la relación es inversa, con un IC = -2,7 para el 2012, mostrando una tendencia pro-pobre.

La cobertura de la vacuna *BCG* está mejor posicionada, con valores cercanos al 100% para el 2012 (Q1 = 96,2%; Q5 = 98,5%). El índice de concentración es casi cero (0,5) que implica una mayor paridad entre ricos y pobres, y un IDP igual a 3,4. El resto de las vacunas tienen niveles menores, pero por encima del 80%, incluyendo la vacuna SRP (antisarapionosa, antirubeolica, antiparotiditis), que tiene una mejor cobertura para los grupos más pobres en el 2012. En cuanto a la inmunización a niños menores de dos años los IC están más cercanos a la paridad demostrando una mayor igualdad con respecto al resto de indicadores.

La cobertura completa de inmunizaciones disminuyó en el 2012, el promedio nacional fue de 74,3% respecto al 2008 (78,6%). El IC es negativo (-0,6) que implica una casi igualdad con cierta inclinación al grupo pobre.

El crecimiento entre 2003 y 2012 de la suplementación con vitamina A, a niños menores de 5 años, con un incremento de 10,3% en el promedio nacional, presenta un valor atípico para el año 2008 (24,6%), muy alejado de los otros dos valores promedio.

La provisión con suplementos de hierro, sufrió un giro completo, incrementando el promedio nacional para Q1 desde 54,2% en el 2008 hasta 74,4% en el 2012, mientras que en el Q5 hubo disminución de -1.1% en el mismo periodo (figura 4). Sin embargo, los niveles de anemia se incrementaron tanto en los promedios nacionales como en los promedios de la mayoría de los grupos de riqueza.

El otro indicador de acción curativa es el tratamiento con rehidratación oral (TRO) y la continuidad en la alimentación del niño menor de 5 años con diarrea, duplicando el promedio en el periodo 2003 – 2012, mostrando alta cercanía con lo que se considera igualdad en todos los grupos de pobreza y zona de residencia.

### Índice Compuesto de Cobertura, según quintiles de riqueza

**FIGURA 2.**
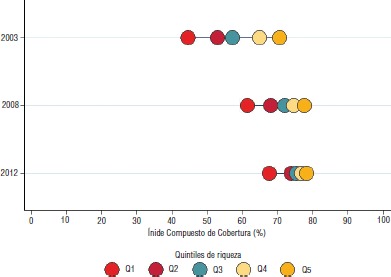
Índice compuesto de cobertura (ICC), estratificado según quintiles de riqueza calculado para las tres encuestas

El Índice Compuesto de Cobertura (ICC), calculado para las tres encuestas creció entre el 2003 y el 2012 (figura 2), se realizó una comparación de las medidas absolutas (IDP) según quintiles de riqueza entre encuestas, la disminución en la pendiente muestra que las desigualdades han decrecido en el transcurso del periodo de estudio. En la encuesta 2003 la desigualdad absoluta era de 30,5, para el 2008 disminuyó a 19,7 y en el 2012 tenía un valor de 14,8, lo que representa 17,8 puntos porcentuales de disminución; estos datos sugieren que la cobertura ha crecido en los grupos más pobres.

**FIGURA 3.**
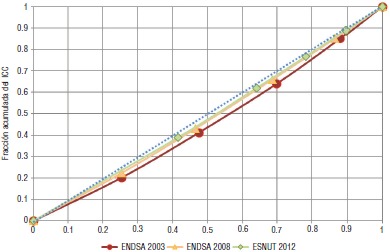
Variación del IC del índice de cobertura compuesta para las tres encuestas

Las variaciones en el índice de concentración son positivas para todas las encuestas (figura 3), la tendencia se mantiene pro-rica, sin embargo, estos valores se acercan más a la diagonal, que significa que hay una tendencia a la disminución de las desigualdades, con una mejor cobertura en los grupos más pobres y en el área rural.

***Fuente:*** elaboración propia, datos ENDSA 2003, 2008 y ESNUT 2012.

**Nota:** cuanto más larga es la línea entre los dos grupos, mayor es la desigualdad absoluta; los puntos representan las coberturas promedio en cada quintil de riqueza, los quintiles Q1(círculo rojo) es el 20% más pobre y Q5(círculo dorado) es el 20% más rico.

### Indicadores de impacto: desnutrición crónica y anemia

Los datos analizados sugieren una disminución en la desnutrición crónica en niños menores de 5 años, con una relación inversa en el indicador de riqueza, con una desigualdad relativa de -20,9 y una desigualdad absoluta de -31,0, que es común dado que el retraso en crecimiento afecta a los grupos más pobres.

En este indicador se pudo ver el descenso entre periodos para todos los quintiles, habiendo logrado una disminución en el promedio nacional de 9,2% entre los periodos 2008 y 2012.

**FIGURA 4.**
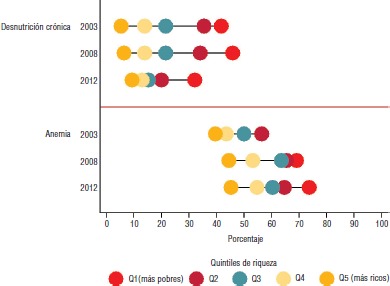
Indicadores de desnutrición crónica y anemia estratificados según quintiles de riqueza calculado para las tres encuestas

La anemia, presentó un comportamiento inverso, la brecha se fue ampliando y los porcentajes se incrementaron en vez de disminuir pese a las intervenciones de provisión de chispitas nutricionales como parte de los suplementos de hierro (figura 4).

***Fuente:*** elaboración propia, datos ENDSA 2003, 2008 y ESNUT 2012.

**Nota:** la línea punteada representa la igualdad, cuanto mayor es el área entre la curva y la diagonal mayor es la desigualdad; los dos últimos periodos prácticamente se solapan.

## DISCUSIÓN

En Bolivia, existen muchas dificultades para documentar los cambios en los patrones de desigualdad, debido a la escasez de información publicada que contenga datos necesarios para realizar procesos de comparación y seguimiento. Las encuestas estandarizadas que se realizan son esenciales para monitorear el progreso e identificar tendencias en las desigualdades ya que no se cuenta con una manera rutinaria de medir estos cambios como en otros países, por ejemplo, Colombia, donde se ha implementado un observatorio de desigualdades en la salud (19).

En los últimos años se ha producido un aumento sustancial del financiamiento público de programas sociales y la adopción de importantes políticas e iniciativas estratégicas relacionadas con la salud (19,20). Los países de LAC tienen un enfoque distinto (21), combinando estrategias de reducción de la pobreza con expansión de servicios integrales de atención primaria de salud, por ejemplo, el Programa Saúde da Família en Brasil (22).

***Fuente:*** elaboración propia, datos ENDSA 2003, 2008 y ESNUT 2012.

**Nota:** cuanto más larga es la línea entre los dos grupos, mayor es la desigualdad absoluta, los puntos representan las coberturas promedio en cada quintil de riqueza; los quintiles Q1(círculo rojo) es el 20% más pobre y Q5(círculo dorado) es el 20% más rico; para el indicador de anemia 2003, los quintiles Q1 y Q2 se solapan por valores muy cercanos.

En Bolivia, en los últimos 10 años se han implementado diferentes estrategias orientadas a mejorar el acceso en salud, principalmente en atención primaria en salud con la creación de programas como Salud Familiar Comunitaria Intercultural (SAF- CI) y Mi Salud (23), estrategias dirigidas a acercar los sistemas de salud a la población y que probablemente ha contribuido en la disminución de las desigualdades, especialmente en indicadores como la atención prenatal por personal de salud.

Las desigualdades en la cobertura de inmunizaciones, la provisión de chispitas nutricionales como suplemento de hierro, y la provisión de vitamina A se han reducido entre quintiles de riqueza. Consideradas como intervenciones de supervivencia infantil, han recibido mayor atención como estrategia para aumentar la cobertura y mejorar la eficiencia de la entrega de servicios, algunas de estas con mayores logros que otras.

Los datos sugieren que no se han logrado mejoras o se ha retrocedido en el uso de métodos de planificación familiar y uso de cualquier método de anticoncepción, una posible explicación se presenta a través de la presencia del Bono Juana Azurduy creado el año 2009 (24), este es un programa de transferencias condicionadas a mujeres embarazadas y niños menores de 2 años, que reciben montos en efectivo diferenciados por control prenatal y de crecimiento hasta los 2 años en el sistema público de salud.

El análisis de la desnutrición se basa en trabajos anteriores presentados en Lancet Nutrition Series 2013 (25). Los intervalos amplios entre las encuestas ayudan a maximizar la probabilidad de evidenciar cambios en los patrones de desigualdad, asumiendo que estos patrones no cambiarían en el corto plazo dada la larga duración del proceso de disminución de desnutrición (25, 26). En la desnutrición crónica en niños menores de 5 años*,* los datos sugieren que las desigualdades han disminuido; probablemente debido a que durante los últimos 20 años en Bolivia se ha trabajado bastante en este tema, con la participación de varias agencias internacionales que han apoyado al sistema de salud para revertir los niveles de desnutrición.

Los datos sugieren que, en términos de igualdad respecto de la desnutrición, el peor año fue el 2008; para el año 2012 hubo cambios hacia la disminución de la brecha, observándose las mayores reducciones de las desigualdades absolutas y relativas.

El Índice Compuesto de Cobertura (ICC) calculado muestra que los grupos más pobres se han acercado a los grupos más ricos reduciendo la brecha de desigualdad, esto es debido al incremento en las coberturas, sin embargo, el cambio en los grupos más ricos no es tan acelerado como en los grupos pobres y del área rural, lo que ha creado un límite general al sistema de salud.

El análisis de co-cobertura (13), a través del ICC, podría ayudar a identificar mejor estrategias o intervenciones y si se necesitan acciones adicionales o alternativas para llegar a otros grupos que aún no han sido beneficiados.

Este es un estudio ecológico que facilita la comparación entre las tres encuestas y permitió investigar las diferencias entre los diferentes grupos, sin embargo, se debe considerar la falacia ecológica, que está implícita en el diseño, donde la asociación observada entre variables agregadas no necesariamente se da a nivel individual y la existencia del sesgo de agregación se presenta como limitante en este tipo de estudios (7).

Una de las limitante es que una explicación correlacional con otras variables a partir de datos individuales no se puede tener, pero queda como parte de hipótesis para otro tipo de estudios.

Se debe mencionar que hay otras dimensiones de desigualdades relevantes para el análisis de cobertura de las intervenciones, por ejemplo, la escolaridad de las madres, grupo étnico y más, que no se abordaron en el presente análisis ya que se utilizó el “índice de riqueza”, que cubre otras dimensiones de desigualdades (27,28).

En conclusión, los datos analizados sugieren avances en la reducción de la desigualdad y disminución en la brecha entre Q1 y Q5, sugiriendo que las acciones realizadas han tenido alcance en los grupos más vulnerables.

Se considera haber alcanzado un límite a las coberturas en el grupo Q5, por lo que se hace necesaria la formulación de estrategias de intervención en el sistema de salud que apunten tanto a la mejora de la calidad como al acceso.

La presencia de bonos con transferencia monetaria podría haber afectado las coberturas en cuanto a uso de métodos anticonceptivos, por lo que se debe investigar con mayor detenimiento esta hipótesis.

Las estrategias de apoyo de suplementación con hierro deben ser revisadas. Los procesos de vacunación han alcanzado niveles ponderables en cuanto a cobertura, siendo este grupo de indicadores los más igualitarios respecto a los quintiles de riqueza y área de residencia.

Estos hallazgos sirven de base para debatir estrategias en salud, con el fin de reducir las desigualdades y muestran la importancia de la implementación de mecanismos que realicen una recopilación regular de datos.

Los principales logros en la reducción de las desigualdades se han obtenido en el periodo 2003 – 2008, siendo mucho menor en el periodo 2008 - 2012.

### Declaración.

Las opiniones expresadas en este manuscrito son responsabilidad del autor y no reflejan necesariamente los criterios ni la política de la *RPSP/PA- JPH* y/o de la OPS

## Footnotes

2 Definiciones de los indicadores en el anexo 1
